# Screening and chromosome localization of two cotton BAC clones

**DOI:** 10.3897/CompCytogen.v10i1.5304

**Published:** 2016-01-22

**Authors:** Xinglei Cui, Fang Liu, Yuling Liu, Zhongli Zhou, Chunying Wang, Yanyan Zhao, Fei Meng, Xingxing Wang, Xiaoyan Cai, Yuhong Wang, Renhai Peng, Kunbo Wang

**Affiliations:** 1 State Key Laboratory of Cotton Biology (China)/Institute of Cotton Research of Chinese Academy of Agricultural Science, Anyang, Henan, 455000,China; 2 Anyang Institute of Technology, Anyang, Henan, 455000, China

**Keywords:** Cotton, BAC, FISH, cytological marker, microcolinearity

## Abstract

Two bacterial artificial chromosome (BAC) clones (350B21 and 299N22) of Pima 90-53 cotton [*Gossypium barbadense* Linnaeus, 1753 (2n=4x=52)] were screened from a BAC library using SSR markers. Strong hybridization signals were detected at terminal regions of all A genome (sub-genome) chromosomes, but were almost absent in D genome (sub-genome) chromosomes with BAC clone 350B21 as the probe. The results indicate that specific sequences, which only exist at the terminal parts of A genome (sub-genome) chromosomes with a huge repeat number, may be contained in BAC clone 350B21. When utilizing FISH with the BAC clone 299N22 as probe, a pair of obvious signals was detected on chromosome 13 of D genome (sub-genome), while strong dispersed signals were detected on all A genome (sub-genome) chromosomes. The results showed that peculiar repetitive sequence, which was distributed throughout all A genome (sub-genome) chromosomes, may exist in BAC clone 299N22. The absence of the repetitive sequences, which exist in the two BAC clones, in D genome may account for the genome-size variation between A and D genomes. In addition, the microcolinearity analysis of the clone 299N22 and its homologous region on *Gossypium raimondii* Ulbrich, 1932 chromosome 13 (D_5_13) indicated that the clone 299N22 might come from A sub-genome of sea island cotton (*Gossypium barbadense*), and a huge number of small deletions, illegitimate recombination, translocation and rearrangements may have occurred during the genus evolution. The two BAC clones studied here can be used as cytological markers but will be also be helpful to research in cotton genome evolution and comparative genomics.

bacterial artificial chromosome

## Introduction

Cotton (*Gossypium* Linnaeus, 1753) provides an excellent model system for studies on polyploidization, genomic organization, and genome-size variation (Wang et al. 2010). The genus of cotton is known to be cultivated in over 100 countries and has been classified into eight diploid (2n=2x=26) genomic groups: A, B, C, D, E, F, G, K, and one allotetraploid (2n=4x=52) genomic group: AD (Percival et al. 1999). Approximately 5 MYA (million years ago) A and D genome diploids diverged, then later became reunited with allopolyploid formation 1–2 MYA (Cronn et al. 2002; Senchina et al. 2003). The latest research shows that the genome size of an A genome species is larger than that of a D genome species (Wang et al. 2012; Li et al. 2014). Many influential factors, such as polyploidization (Wendel 2000), transposable element ampliﬁcation (Bennetzen 2002; Kidwell et al. 2002; Piegu et al. 2006), tandem repeat expansion (Ellegren et al. 2002; Morgante et al. 2002), gene duplication (Zhang 2003), organellar transfer to the nucleus (Shahmuradov et al. 2003), and intron size expansion (Deutsch et al. 1999; Vinogradov et al. 1999) are thought to be collectively responsible for the genome-size variation (Grover et al. 2007). Accumulation of different transposable elements classes among different genomes was thought to be the most important reason (Hawkins et al. 2006). The studies on genome-size differences between A and D genomes will help in understanding cotton evolution as well as facilitating genetic improvement of cotton.

The introduction of fluorescence *in situ* hybridization (FISH), involving hybridization of labeled DNA probes to cytological targets, such as metaphase chromosomes, interphase nuclei, and extended DNA fibers, marked the beginning of a new era for studies on chromosome structure and function. Modern methodologies and modifications, such as the development of probes from specificity for highly repeated sequences to single-copy sequence (Desel et al. 2001; Zhu et al. 1999), and from single-colored probes to multiple-colored probes (Tang et al. 2009), have all been designed to optimize the probe detection sensitivity. Nowadays, FISH is a versatile and accurate tool for chromosome localization of sequences (Gomez et al. 1997), cytogenetic map construction (Sun et al. 2013; Han et al. 2011; Cui et al. 2015), genome structure study (Zhao et al. 2011; Wang et al. 2001a), genome evolution (Wu et al. 2013), and comparative genomics study (Gan et al. 2013).

Eukaryotic genomes, with rare exceptions, are replete with interspersed repetitive DNAs, of which most are transposable elements (Feschotte 2008). Large-scale DNA sequencing has revealed that genome size is highly correlated with transposable element content (Oliver et al. 2013). The genomes of *Gossypium arboretum* Linnaeus, 1753 and *Gossypium raimondii* Ulbrich, 1932 have been sequenced and assembled, the comparison between the two genomes showed the transposable elements, especially LTR, activities substantially contributed to the twofold genome-size variation (Wang et al. 2012; Li et al. 2014). In this study, two BAC clones with genome-specific repetitive sequences (350B21 and 299N22) were localized and microcolinearity of BAC clone 299N22 and its homologous region on chromosome D_5_13 was analyzed.

## Material and methods

### Materials

The plant materials were obtained from National Wild Cotton Nursery in Hainan Island, China, sponsored by the Institute of Cotton Research of Chinese Academy of Agricultural Sciences (CRI-CAAS). They are also conserved in the greenhouse at CRI-CAAS’ headquarter in Anyang City, Henan Province, China.

Chromosome-specific BAC clones (Wang et al. 2007) used to identify the individual chromosomes were kindly provided by Prof. Tianzhen Zhang (Nanjing Agricultural University, China).

The *Gossypium raimondii* genome sequence was downloaded from the sequenced genome of land plants in Phytozome (http://www.phytozome.net). The *Gossypium arboreum* genome sequence was downloaded from Cotton Genome Project (CGP: http://cgp.genomics.org.cn).

### Screening of BAC library

Pima 90–53 (*Gossypium barbadense*) BAC library screened in this paper was kindly provided by Prof. Zhiying Ma (Hebei Agricultural University, China). The simple sequence repeat (SSR) markers were selected from 3 genetic maps (Table 1) (Nguyen et al. 2004; Zhao et al. 2012; Han et al. 2006) and used to screen the BAC library. To facilitate PCR screening, a rapid method of screening BAC libraries was used to obtain positive BAC clones (Cheng et al. 2012). First, one-dimensional pools (plate pools) were made; 384 clones were pooled together on a same plate. Then, bacterial colony PCR was used to screen one-dimensional pools. Secondly, two-dimensional pools (line pools) were made and used to screen, in each of which 24 clones in a same line were pooled together. Thirdly, each clone was screened for the target DNA. Bacterial colony PCR was carried out with 1µL of Bacterial colony template in the presence of 0.5 µL of dNTPs (10mM), 0.5 U Taq DNA polymerase, 1.0 µL 10×Reaction buffer and 0.5 µL of each primer, for a final volume of 10 µL. Following initial denaturation at 95 °C for 3 min, 30 cycles of 94 °C for 45 s, annealing temperature for 45 s and 72 °C for 1 min was performed. PCR products were separated by 0.8% polyacrylamide gel electrophoresis.

**Table 1.** SSR markers and their genetic maps.

SSR marker	Genetic map of cotton
NAU1215	Han et al. (2006) Theor Appl Genet
CIR342	Han et al. (2006) Theor Appl Genet
NAU1023	Han et al. (2006) Theor Appl Genet
NAU1201	Han et al. (2006) Theor Appl Genet
NAU3022	Zhao et al. (2012) BMC Gnomics
NAU3384	Zhao et al. (2012) BMC Gnomics
NAU5100	Zhao et al. (2012) BMC Gnomics
CIR096	Nguyen et al. (2004) Theor Appl Genet

### DNA probe preparation

The BAC clone DNA was isolated using a standard alkaline extraction (Sambrook et al. 2002). The chromosome-specific BAC clones were labeled with digoxigenin-dUTP via nick translation, whereas the screened BAC clones were labeled with biotin-dUTP via nick translation, according to the instructions of the manufacturer (Roche Diagnostics, USA).

### Chromosome preparation and FISH

Mitotic chromosome preparation and FISH procedures were conducted using a modified protocol (Wang et al. 2001b). Biotin-labeled and digoxigenin-labeled probes were detected by avidin-fluorescein (green) and anti-digoxigenin-rhodamine (red) (Roche Diagnostics, USA), respectively. Chromosomes were counterstained by 4’,6-diamidino-2-phenylindole (DAPI) in antifade VECTASHIELD solutions (Vector Laboratories, Burlingame, CA). The concentration of block DNA (genomic DNA) was 200 times that of the chromosome-specific BAC DNA. The hybridization signals were observed using a fluorescence microscope (Leica MRA2) with a charge-coupled device (CCD) camera. Final image adjustments were performed using Adobe Photoshop CS3 software.

### BAC clone sequencing and microcolinearity analysis

Both BAC clone 350B21 and 299N22 were outsourced to a biological company for sequencing. The sequences of BAC clones were used as query sequences to search for its homologous regions using BLASTN algorithms against A_2_ genome and D_5_ genome. Microcolinearity analysis of homologous regions was achieved using software CIRCOS.

## Results

### Identification and selection of cotton BAC clones

A total of 192 plate pools (73728 BAC clones, nearly covering *Gossypium barbadense* genome 3 times) were constructed and screened using bacterial colony PCR. Nineteen positive BAC clones were identified (Table 2) and selected to be probes for FISH. Seventeen clones, which showed ambiguous FISH signals or no FISH signal on *Gossypium barbadense* mitotic metaphase chromosomes, were discarded. BAC clones 350B21 and 299N22, which showed obvious characteristic signals on *Gossypium barbadense* mitotic metaphase chromosomes, were selected for further study.

**Table 2.** Screened clones of Pima 90-53 BAC library.

SSR markers	Screened clones from BAC library
NAU1215	300N10
CIR342	268E2; 268K2
NAU1023	311A4; 311A11
NAU1201	299N22; 323O3; 317K24; 185N14
NAU3022	30A18; 106P24
NAU3384	328L13
NAU5100	389J15; 376M12; 311M1
CIR096	399A22; 162G3; 350B21; 342O11

### Localization of BAC 350B21

**Figure 1.**
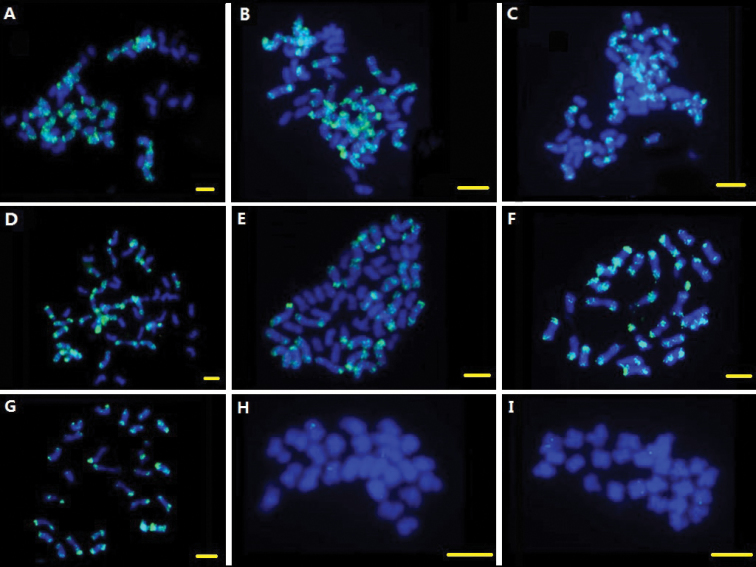
The FISH images of BAC clone 350B21(green) hybridized to mid-mitotic chromosomes in different *Gossypium* species, Bar: 5 µm. **A** *Gossypium hirsutum* (A_1_D_1_, 2n=4x=52) **B** *Gossypium barbadense* (A_2_D_2_, 2n=4x=52); **C** *Gossypium tomentosum* (A_3_D_3_, 2n=4x=52) **D** *Gossypium mustelinum* (A_4_D_4_, 2n=4x=52) **E** *Gossypium darwinii* (A_5_D_5_, 2n=4x=52) **F** *Gossypium arboretum* (A_1_, 2n=2x=26) **G** *Gossypium herbaceum* (A_2_, 2n=2x=26) **H** *Gossypium thurberi* (D_1_, 2n=2x=26) **I** *Gossypium raimondii* (D_5_, 2n=2x=26).

Obvious signals were detected on terminal parts of all *Gossypium barbadense* Linnaeus, 1753 (A_2_D_2_, 2n=4x=52) A sub-genome chromosomes with BAC clone 350B21 as probe. And signals were alike when using four other tetraploid species [*Gossypium hirsutum* Linnaeus, 1753 (A_1_D_1_, 2n=4x=52), *Gossypium tomentosum* Nuttall ex Seemann, 1865 (A_3_D_3_, 2n=4x=52), *Gossypium mustelinum* Miers ex Watt, 1907 (A_4_D_4_, 2n=4x=52), *Gossypium darwinii* Watt, 1907 (A_5_D_5_, 2n=4x=52)] mitotic metaphase chromosomes as target DNAs. Then, mitotic metaphase chromosomes of two A genome species [*Gossypium arboretum* (A_1_, 2n=2x=26), *Gossypium herbaceum* Linnaeus, 1753 (A_2_, 2n=2x=26)] were used as target DNAs and obvious signals were detected at terminal parts of all the chromosomes. On the contrast, no obvious signal, except two pair of weak signals, was detected on chromosomes of two D genome cotton species [*Gossypium thurberi* Todaro, 1878 (D_1_, 2n=2x=26) and *Gossypium raimondii* (D_5_, 2n=2x=26)]. The signals were alike between A genomes and A sub-genomes as well as D genomes and D sub-genomes (Fig. 1).

### Localization of BAC 299N22

**Figure 2.**
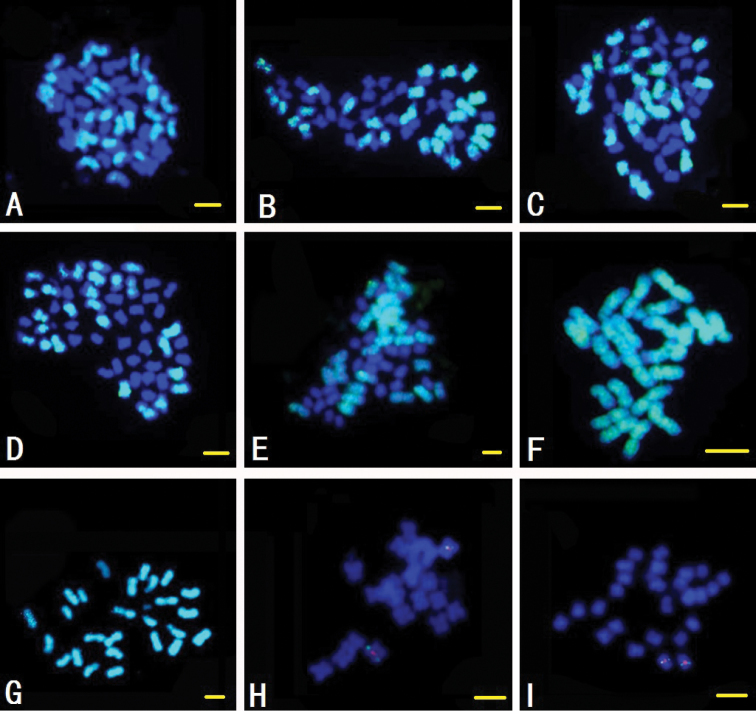
The FISH images of BAC clone 299N22 (green) hybridized to mid-mitotic chromosomes different *Gossypium* species, Bar: 5 µm. **A** *Gossypium hirsutum* (A_1_D_1_, 2n=4x=52) **B** *Gossypium barbadense* (A_2_D_2_, 2n=4x=52) **C** *Gossypium tomentosum* (A_3_D_3_, 2n=4x=52) **D** *Gossypium mustelinum* (A_4_D_4_, 2n=4x=52) **E** *Gossypium darwinii* (A_5_D_5_, 2n=4x=52) **F** *Gossypium arboretum* (A_1_, 2n=2x=26); **G** *Gossypium herbaceum* (A_2_, 2n=2x=26) **H** *Gossypium thurberi* (D_1_, 2n=2x=26) **I** *Gossypium raimondii* (D_5_, 2n=2x=26). Red: the signal of chromosome-specific BAC clone for chromosome D_1_13, D_5_13.

Obvious disperse signals were detected on all A sub-genome chromosomes of tetraploid species [*Gossypium hirsutum* (A_1_D_1_, 2n=4x=52), *Gossypium barbadense* (A_2_D_2_, 2n=4x=52), *Gossypium tomentosum* (A_3_D_3_, 2n=4x=52), *Gossypium mustelinum* (A_4_D_4_, 2n=4x=52), *Gossypium darwinii* (A_5_D_5_, 2n=4x=52)] with BAC clone 299N22 as probe. When mitotic metaphase chromosomes of two A genome species [*Gossypium arboretum* (A_1_, 2n=2x=26), *Gossypium herbaceum* (A_2_, 2n=2x=26)] were used as target DNAs, obvious signals were detected on all the chromosomes, while only a pair of obvious signals was detected on chromosome 13 of two D genome cotton species [*Gossypium thurberi* (D_1_, 2n=2x=26) and *Gossypium raimondii* (D_5_, 2n=2x=26)]. The relative position of FISH signals on chromosome D_5_13 was measured to be about 62.4FL (FL: the percentage of the distance from the FISH site to the end of the short arm relative to the total length of the chromosome) after measuring more than 10 cells with clear chromosome spreads (Fig. 2).

### Analysis of BAC clones sequences and microcolinearity

**Figure 3.**
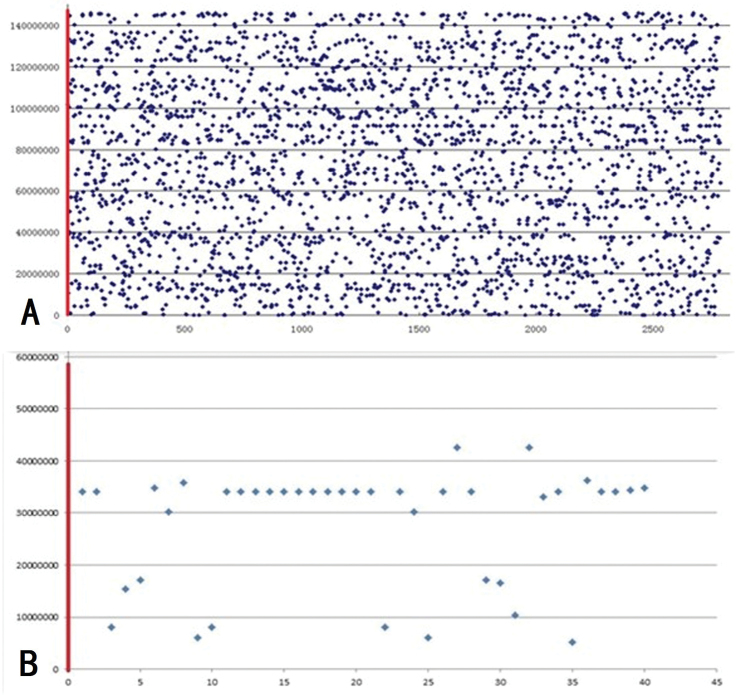
The distribution of BAC 299N22 clone on chromosomes. X-coordinate indicates the length of chromosome, y-coordinate indicates the hits of sequence alignment of BAC clone 299N22 and chromosomes. **A** the result of BLASN, with chromosome A_2_02 as database **B** the result of BLASTN, with chromosome D_5_13 as database.

**Figure 4.**
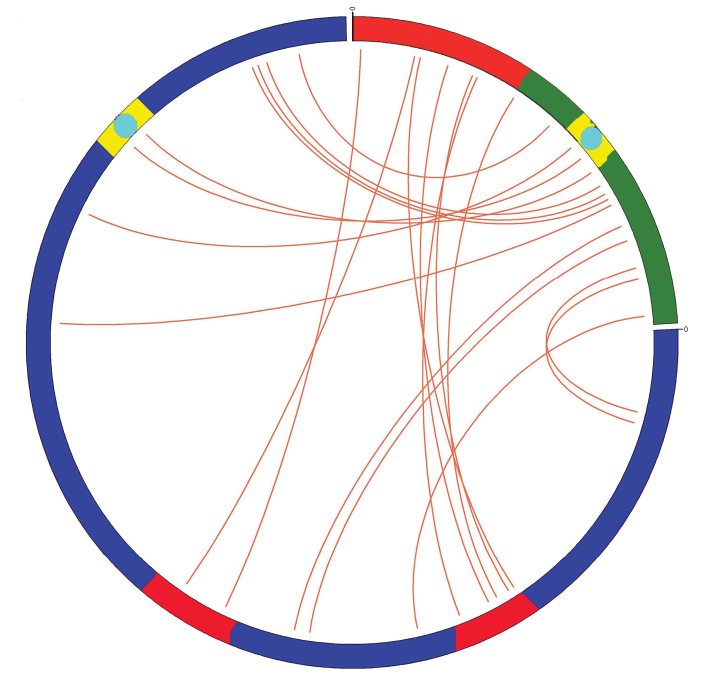
Microcolinearity of the BAC 299N22 and its homologous fragment on chromosome D_5_13 of *Gossypium raimondii*. Lower-left is BAC 299N22, top-right is its homologous fragment on chromosome D_5_13 of *Gossypium raimondii*. Red: gene 013G130100, yellow: 013G130200, light blue: 4 SSR markers.

Sequencing of BAC clone 350B21 failed, as too many simple repeat sequences existed in the BAC clone. A new lineage-specific LTR family, which accounted for about 35% of A_2_ genome while being absent in D_5_ genome, was identified analyzing the sequence of BAC 299N22. The sequence of BAC clone 299N22 was used as query sequence to search for its homologous regions using BLASTN algorithms against A_2_ genome (*Gossypium arboretum*) and D_5_ (*Gossypium raimondii*) genome, respectively. When A_2_ genome was used as a database, multiple dispersedly distributed hits on all chromosomes of A_2_ genome were obtained (Fig. 3A), so the homologous region of BAC 299N22 in A_2_ genome was not identified. When D_5_ genome was used as a database, similar sequences were only detected in chromosome 13, and the density was obviously higher at the region of 34067000bp—34098000bp (58.41% of chromosome D_5_13, the position was almost the same as FISH result) than that of other regions of D_5_13 chromosome. When the E value was set lower, the hits were only found in that region (Fig. 3B). Therefore, the 31kb region on chromosome D_5_13 was thought to be the homologous region of BAC 299N22. Using the CIRCOS software analysis, the microcolinearity of BAC clone 299N22 and its homologous region on chromosome D_5_13 proved to be poor. The orders of the highly conserved fragments showed discrepancies, even the orientations of some highly conserved fragments were different. According to cotton SSR primer sequence information on NCBI and *Gossypium raimondii* genome annotation information, 4 SSR markers, NAU1201, NAU1141, HAU3220, and MON_CGR5697, and 2 genes 013G130100 and 013G130200, were contained in the pair of homologous regions (Fig. 4). However, the distance between the two genes in BAC clone 299N22 was much longer than that on D_5_13, and gene 013G130100 in BAC clone 299N22 was divided into two segments. The results also showed special sequences, which do not exist in D genome, repeated a huge number of times in A genome, exist in BAC 299N22.

## Discussion

### New cytological markers

Chromosome identification is the foundation of research on plant genetics, evolution and genomics. Conventional individual chromosome identification is mainly based on analyzing chromosomal relative lengths and arm ratios, and, as a result, is very difficult and inaccurate when identifying chromosomes small and similar. Therefore finding suitable molecular cytogenetic markers becomes very necessary for the unambiguous identification of individual chromosomes. FISH is a reliable cytological technique for chromosome identification, and has been adapted successfully to identify the chromosomes for many plant species, including rice (Cheng et al. 2001), potato (Dong et al. 2000), sorghum (Kim et al. 2005) and so on. A set of chromosome-specific BAC clones for *Gossypium hirsutum* chromosomes identification has been developed and was applied successfully in many cotton species (Wang et al. 2007; Wang et al. 2008; Gan et al. 2011; Gan et al. 2012). In this study, BAC clone 299N22 could be a new cytological marker for chromosome 13 of D genome (sub-genome), and its cytogenetic position was measured to be approximately 62.4 FL. As BAC clone 299N22 showed well-distributed repetitive signals on all A genome (sub-genome) chromosomes, it also could be used as a cytological marker for identifying A genome (sub-genome) chromosomes. BAC clone 350B21, which showed repetitive signals at the terminal regions of all A genome (sub-genome) chromosomes could be used as a cytological marker for identifying or labeling terminal regions of all A genome (sub-genome) chromosomes. The addition of these new cytological markers will facilitate the study of cotton genomics and evolution.

### Cotton A genome (sub-genome) has unique repetitive sequences

Repetitive DNA sequences form a large portion of the genomes of eukaryotes, indicating a major contributor to variation in genome size among organisms of similar complexity (Charlesworth et al. 1994). The genus *Gossypium*, which provides a facile system for investigating the genomic organization and evolution, also has a high content of repetitive sequences in its genome. Different types of repeat sequences accounted for as much as 68.5% of the *Gossypium arboreum* genome and approximately 57% of the *Gossypium raimondii* genome, respectively. And most of the repetitive sequences are long terminal repeat (LTR) retrotransposons (Wang et al. 2012; Li et al. 2014).

When using BAC clone 350B21 as a probe, strong signals were detected at the terminal parts of all chromosomes of A genome (sub-genome), while being absent on D genome (sub-genome) chromosomes. The results may indicate that special repetitive sequences in BAC clone 350B21 have a bias of insertion sites at terminal parts of A genome (sub-genome) chromosomes. Another kind of repetitive sequence exists in BAC clone 299N22 showed well-distributed dispersed signals on all A genome (sub-genome) chromosomes. These unique repetitive sequences may be the major reason for the genome-size difference between A genome and D genome.

A new LTR family, which accounts for about 35% of A_2_ genome while almost being absent in D_5_ genome, was identified analyzing the sequence of BAC clone 299N22. The LTR family was inserted randomly along each chromosome in *Gossypium arboretum* genome, and was different from any reported repetitive sequences in cotton (Hawkins et al. 2006). As the LTR family accounts for so much of A genome, it should be different from any sequence reported by Zhao et al (1995) and Hanson et al (1998). The identification of the new LTR family will facilitate understanding of the differences between the two genomes. Sequencing of BAC clone 350B21 failed as too many simple repeat sequences existed in the BAC clone. This indicates that the terminal regions of A genome (sub-genome) chromosomes may be replete with simple repeat sequences. Their absence in D genome (sub-genome) indicates that they may appear after the divergence of A, D genomes and contributed to the genome-size difference between the two genomes. The similarity of signals in D genome and D sub-genome suggests that the repetitive sequences in the two BAC clones may not occur colonization after polyploidization event, this indicate they may turned to be silent before the polyploidization event.

### Many factors contributed to genome-size evolution.

Many factors are thought to be responsible for the genome-size variation. The analysis of *AdhA* and *CesA* regions of different cotton genomes indicated that many forces operated collectively among genomic regions to reflect genome-size evolution (Grover et al. 2007). The microcolinearity analysis, comparative analysis of homologous sequences from different genomes, is a method of comparative genomics research for studying and speculating upon the relationships between genomes and evolution patterns. In the present study, the homologous region of BAC clone 299N22 on chromosome D_5_13 was obtained using bioinformatics analysis. As the sequence of BAC clone 299N22 is much longer than its homologous region on chromosome D_5_13, BAC clone 299N22 was thought to be from A sub-genome of *Gossypium barbadense*. Microcolinearity analysis of the homologous regions showed that the orders of the most highly conserved fragments were different, even the orientations of some highly conserved fragments was different, which may indicate that a large number of translocations, inversions, and segmental rearrangements occurred during evolution. The analysis showed the length of gene parts appeared similar between the homologous regions, while gene-free regions were not. This may provide a hint that the evolution between gene islands or in gene-free regions may be the main reason for the genome-size variations, as previously reported (Grover 2004). The repetitive sequences which were distributed dispersedly on A genome chromosomes were located at the non-genetic regions, and this may indicate that the difference in non-genetic regions may be attributed to the accumulation of repetitive sequences.

## Conclusions

In recent years, many achievements, such as in the study of cytogenetic map construction, genome evolution, and comparative genomics, have been obtained by using BAC-FISH. The repetitive sequences in the two BAC clones showed distribution bias and may be an important reason for the genome-size variation. Analysis of the repetitive sequences will be helpful in the studies on cotton genome evolution and comparative genomics.
